# SREBP1 siRNA enhance the docetaxel effect based on a bone-cancer dual-targeting biomimetic nanosystem against bone metastatic castration-resistant prostate cancer

**DOI:** 10.7150/thno.40489

**Published:** 2020-01-01

**Authors:** Jiyuan Chen, Zhenjie Wu, Weihong Ding, Chengwu Xiao, Yu Zhang, Shen Gao, Yuan Gao, Weimin Cai

**Affiliations:** 1 Department of Clinical Pharmacy and Drug Administration, School of Pharmacy, Fudan University, Shanghai 201203, China; 2 Department of Urology, Changhai Hospital, Second Military Medical University, Shanghai 200433, China; 3 Department of Urology, Huashan Hospital, Fudan University, Shanghai 200040, China

**Keywords:** bone metastatic prostate cancer, fused cell membrane, bone marrow mesenchymal stem cells, docetaxel, SREBP1 siRNA

## Abstract

Until recently, there have been limited options for patients with bone metastatic castration-resistant prostate cancer (BmCRPC) following the failure of or development of resistance to docetaxel (DTX), which is one of the frontline treatments. Sterol regulatory element-binding protein 1 (SREBP1) is reported to regulate abnormal lipid metabolism and to promote the progression and metastasis of prostate cancer (PCa). The siRNA interferes SREBP1 may provide an efficient treatment when combined with DTX.

**Methods**: In this study, lipoic acid (LA) and cross-linked peptide-lipoic acid micelles were cross-linked (LC) for DTX and siSREBP1 delivery (LC/D/siR). Then, cell membrane of PCa cells (Pm) and bone marrow mesenchymal stem cells (Bm) were fused for cloaking LC/D/siR (PB@LC/D/siR). Finally, the synthesized PB@LC/D/siR was evaluated *in vitro* and *in vivo*.

**Results**: PB@LC/D/siR is internalized in PCa cells by a mechanism of lysosome escape. Tumor targeting and bone homing studies are evaluated using bone metastatic CRPC (BmCRPC) models, both *in vitro* and *in vivo*. Moreover, the enhanced anti-proliferation, anti-migration and anti-invasion capacities of DTX- and siSREBP1- loaded PB@LC (PB@LC/D/siR) were observed *in vitro*. Furthermore, PB@LC/D/siR was able to suppress the growth of the tumor effectively with deep tumor penetration, high safety and good protection of the bone at the tumor site. Additionally, the mRNA levels and protein levels of SREBP1 and SCD1 were able to be significantly downregulated by PB@LC/D/siR.

**Conclusion**: This study presented a bone-cancer dual-targeting biomimetic nanodelivery system for bone metastatic CRPC.

## Introduction

Prostate cancer (PCa) is one of the most frequently occurring cancers in males worldwide. The development of castration-resistant prostate cancer (CRPC) after 18~24 months of androgen deprivation therapy is a challenge facing clinical PCa treatment. 1 Moreover, most of these CPRC patients will progress into more aggressive metastatic CRPC (mCRPC), and approximately 90% metastasizes to bone, further developing into bone metastatic CRPC (BmCRPC) with a low survival period. 2,3 Docetaxel (DTX) is a standard-of-care first-line chemotherapy for patients with BmCRPC. 3,4 However, the challenges of chemo-resistance and the high rate of adverse effects have hindered the expansion of clinical applications among currently available DTX-based therapies. 5 Thus, the development of new DTX-based combination therapies with tumor-targeting ability for BmCRPC treatment is urgently needed.

PCa is characterized in part by dysregulation of lipid metabolism, and sterol regulatory element-binding protein (SREBP) is an uncontrollable genetic factor. 6 Abnormal lipid biosynthesis promotes the growth and metastasis of PCa; furthermore, enhanced lipid supply is also associated with bone metastasis of PCa compared with localized PCa. 7 It has been reported that microRNA-185 and -342 can inhibit PCa by repressing the expression of the SREBP metabolic pathway. 8 Moreover, interfering expression of SREBP by siRNA-SREBP1 (siSREBP1) or siRNA-SREBP2 (siSREBP2) could directly downregulate the progression and metastasis of PCa by repressing the PI3K/AKT signaling pathway. 8,9 Moreover, inhibition of PI3K/AKT could increase the sensitivity and abrogate the resistance of DTX in PCa. 10,11 Therefore, the combination of DTX with siSREBP1 might provide an enhanced effect and emerge as a promising strategy for BmCRPC. In order to achieve a better drug combination, it is a good choice to establish a nano-codelivery platform for siRNA delivery with multi-functions such as sustained, controlled and targeted delivery, microenvironment (pH, redox) -sensitive. 12-14

In this study, we developed a biomimetic nano-system (PB@LC) based on a lipoic acid (LA) 15 and cross-linked peptide-lipoic acid micelle 16 cross-linked nano-platform (LC), which was coated by a fused cell membrane of bone marrow mesenchymal stem cells (BMSCs) and prostate cancer cells. Our previous studies demonstrated that cross linked LA nanoparticles (LA-NP) and cross-linked peptide-lipoic acid micelles could provide a targeted delivery effect for chemotherapeutic drugs and gene drugs *in vivo* with reductive sensitive attributes, excellent stability and good safety. 15,16 Cell membrane-coated nanoparticles based on erythrocyte membranes were first developed by Zhang *et al,* 17 which improved the performance of synthetic nanoparticles *in vivo*. After cell membrane cloaking, nanoparticles not only acquire the physiochemical properties of natural cell membranes but also inherit unique biological functions due to the presence of membrane-anchored proteins, antigens, and immunological moieties. 18 Various types of cancer cell membranes have been shown to enhance tumor targetability for nanoparticles with their own cell surface antigens. 19-23 Furthermore, mesenchymal stem cells (MSCs) exhibit priority targeting to inflammation, injury sites, tumors, and especially bone marrow. 24 MSCs can home to the bone marrow niche of myeloma or bone metastatic cancer. 25,26 Additionally, MSC cell membranes also exhibit excellent tumor targeting ability. 27,28 However, to the best of our knowledge, biomimetic nanoparticles coated with PCa cell membranes and fused PCa cell membranes with BMSC cell membranes have not been previously reported. Therefore, PCa cell membranes fused with BMSCs (PBm) might achieve PCa homologous targeting and bone homing abilities, targeting the bone metastatic niche accurately.

**Scheme 1:**
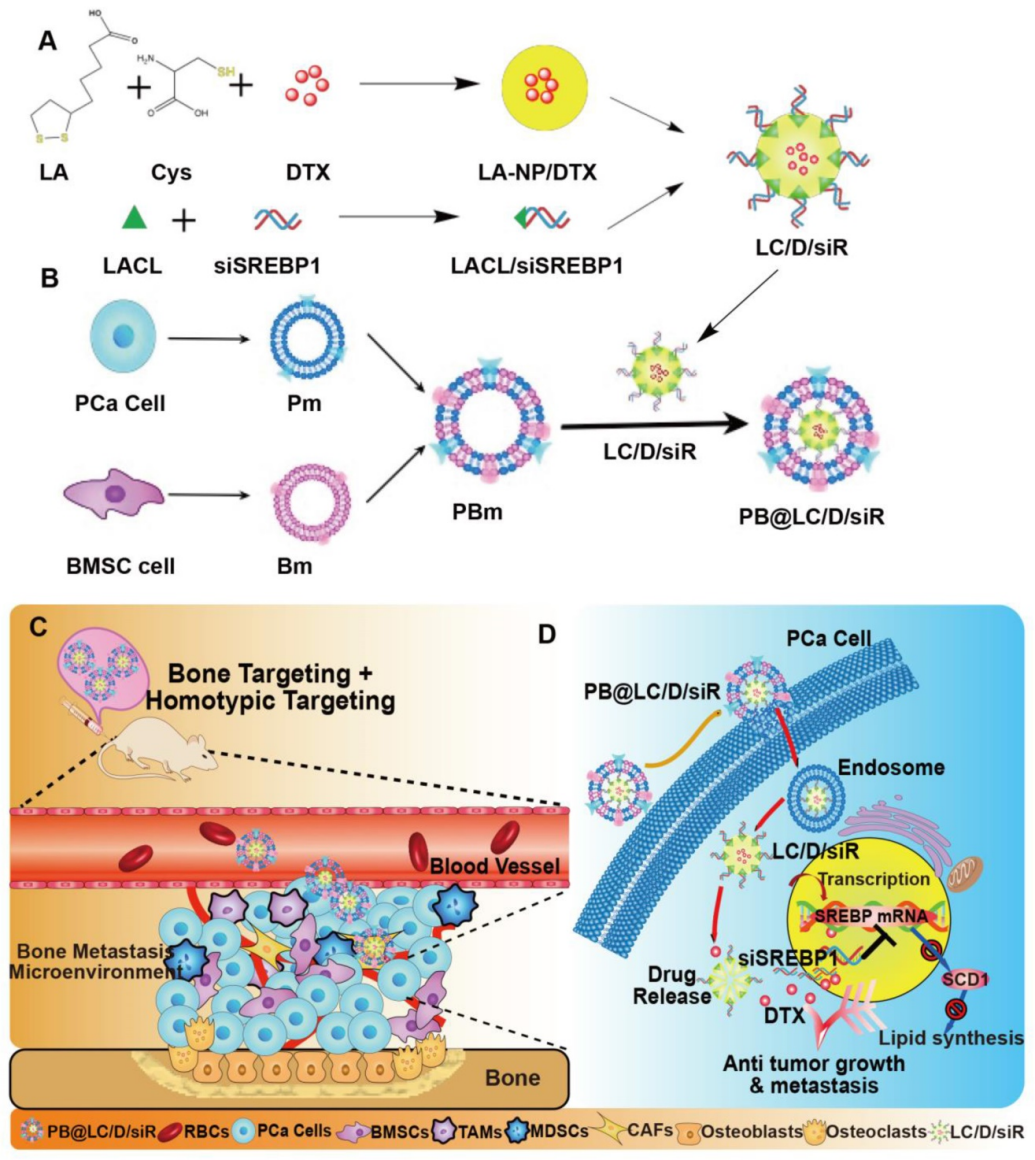
The designation and mechanism of PB@LC/D/siR. A) The preparation of co-loading nanoparticles LC/D/siR. B) The fusion and coating of PBm. C) The schematic illustration of PB@LC/D/siR targeting the microenvironment of BmCRPC based on the fundamental of bone homing and homotypic targeting ability of PBm. D) The effect mechanism of PB@LC/D/siR.

In this study, we hypothesized that PB@LC could be used for the highly targeted delivery of DTX and siSREBP1 to the bone metastatic niche of BmCRPC. To this end, coloading PB@LC/DTX/siSREBP micelles (PB@LC/D/siR) were prepared (Scheme 1). Their enhanced effects and possible mechanisms of the micelles for BmCRPC therapy were further evaluated *in vitro* and *in vivo*.

## Results

### Preparation and characterization of PB@LC

**Figure 1:**
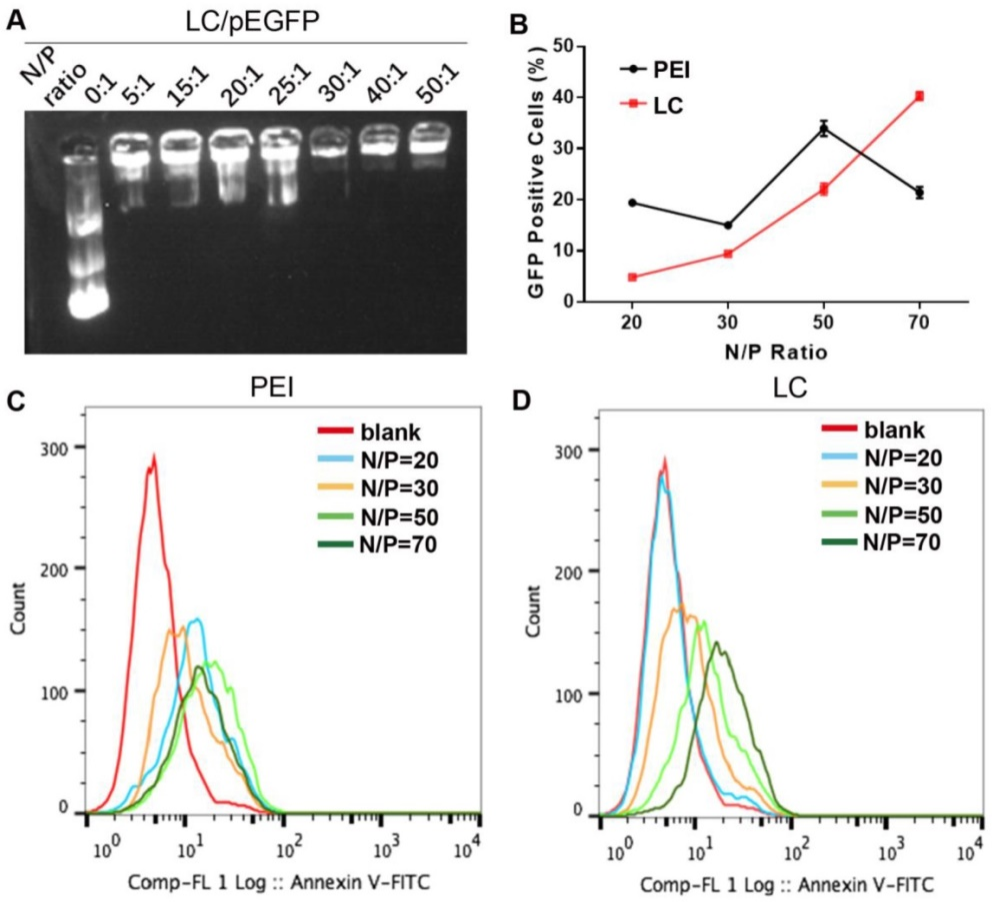
The characterization of gene compression and gene transfection abilities of LC. A) The agarose gel electrophoresis results of different N/P ratios of LC/pEGFP. B-D) The transfection ability of PEI or LC gene carriers in HeK-293T cells at different N/P ratios. B) The statistical results of green fluorescent protein (GFP) positive cells of LC and PEI (n=3, mean ± SD). C-D) The flow cytometry results of C) PEI and D) LC.

LC was prepared as reported previously.^15^,^16^ Both dynamic light scattering (DLS) and transmission electronic microscopy (TEM) revealed a homogeneous hydrodynamic size of ~ 100 nm and spherical shape for LC and LC/D/siR, while the zeta potentials were 26.9 ± 0.1 mV and 20.3 ± 0.1 mV, respectively (n = 3). In addition, LC was stable at 4℃ for over 30 days (Figure S1). As shown in Figure 1A, the gene model drug pEGFP presented tight binding in LC at an N/P ratio of 30 by agarose gel electrophoresis. Moreover, LC exhibited a higher gene transfection capacity than PEI at an N/P ratio of 70 with low cytotoxicity (Figure 1B-D, Figure S2).

**Figure 2:**
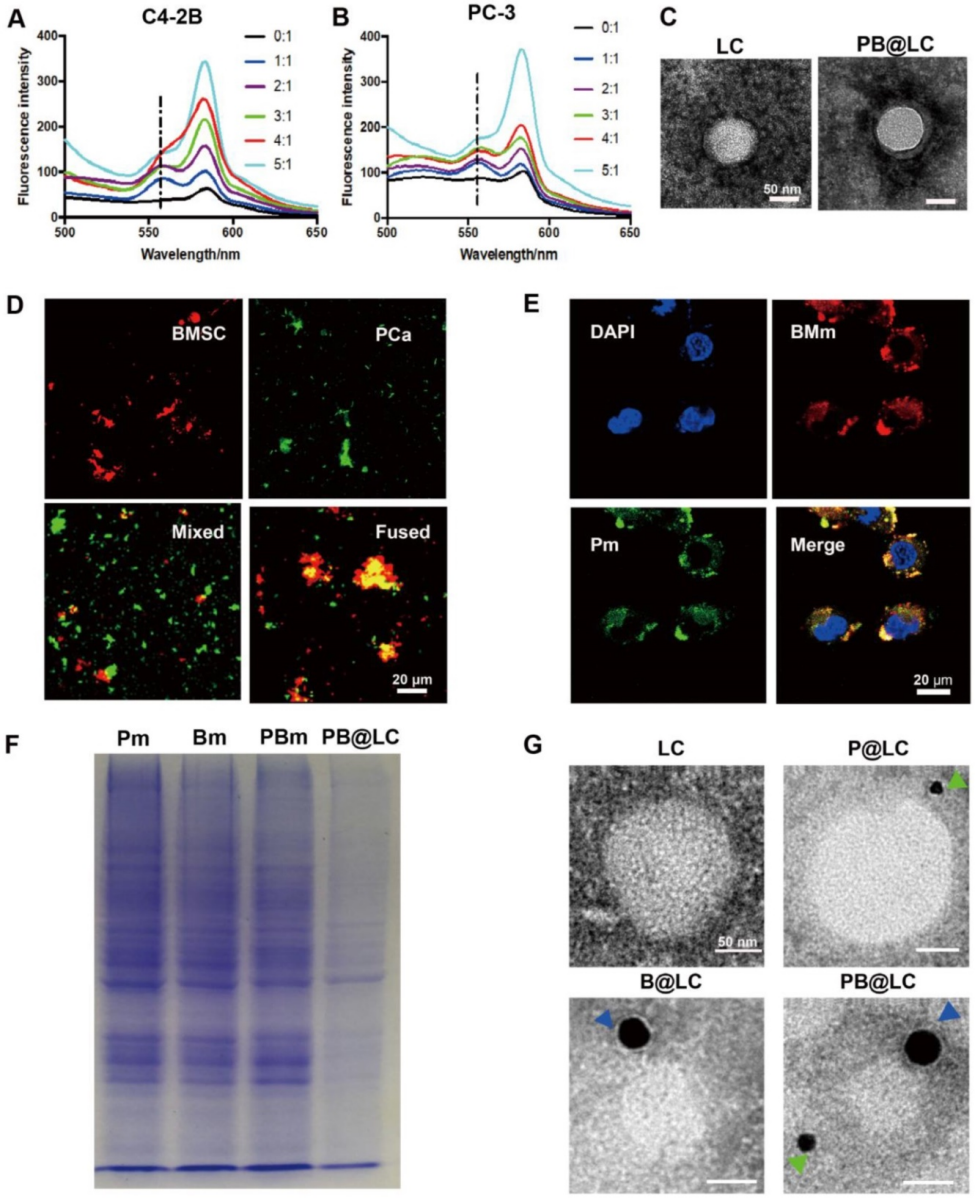
The characterization of PBm and PB@LC. A-B) The fluorescence spectrophotometer results of different membrane protein ratios of 5:1, 4:1, 3:1, 2:1, 1:1, and 0:1 of Bm to Pm. C) The representative TEM images of LC and PB@LC (scale bars = 50 nm). D-E) The representative CLSM images of D) Pm, Bm, Mixture of P Bm, and Fused PBm (scale bars = 20 μm) and E) PB@LC (scale bars = 20 μm). F) The SDS-PAGE gel electrophoresis results of Pm, Bm, PBm, and PB@LC. G) The immunogold TEM images of -11 (green arrows, small gold = 10 nm) and STRO-1 (blue arrows, large gold = 20 nm) probed LC, P@LC, B@LC and PB@LC, followed by negative staining with phosphotungstic acid (scale bars = 50 nm).

Cell membrane-coated biomimetic nanosystems enhanced the *in vivo* performance of current nanosystems, which displayed not only a long circulation time but also good combined biocompatibilities and tumor targeting properties for synthetic nanosystems. 29 To obtain bone targeting capability, we derived BMSC cells from 4-week-old Sprague-Dawley (SD) rats purified by lymphocyte separation solution. 30 The purity of BMSC cells was as high as 90% at the third generation (P3), and the BMSC cell membranes were could be collected until the sixth generation (Figure S3). Meanwhile, PCa cell membranes from two BmCRPC cell lines, PC-3 and C4-2B cells, were derived, respectively. In this study, all cell membranes were prepared through a continuous extrusion method and purified by centrifugation. The fused cell membranes (PBm) were collected by fusion of BMSC cell membranes (Bm) and PCa cell membranes (Pm). The membrane potential of PCa and BMSC cells was approximately -22 mV, and every 10^7^ cells contained 0.6 mg membrane protein (Figure S3). To test for fusion, Bm was dyed with a Förster resonance energy transfer (FRET) dye pair DOPE-RhB/C6-NBD. As the amount of Pm increased, there was a recovery of fluorescence at an emission wavelength of approximately 560 nm, weakening the FRET effect of DOPE-RhB/C6-NBD in Bm. A 1:1 membrane protein weight ratio of Pm to Bm was used for further study (Figure 2A,B). In addition, a 5:1 weight ratio of LC to PBm was produced with optimal particle properties (Figure S4). The size of PB@LC was 92.7 nm after coating, which was approximately 10 nm larger than LC. The potential of PB@LC was -22.0 mV, which was similar to the cell membrane potential (Figure S1). In Figure 2C, a classic core-shell structure of PB@LC could be clearly identified through TEM images. The coverage rate of Pm-coated LC (P@LC) was approximately 90% by the bicinchoninic acid (BCA) method. Additionally, PB@LC was stable in 1×PBS and double-distilled water for 18 days with good biocompatibilities at concentrations as high as 0.4 mg/mL (Figure S5).

To further demonstrate the fusion, Bm and Pm were labeled with a red dye DiR and a green dye DiO, respectively. The resultant materials were mixed or fused Pm/Bm for confocal laser scanning microscopy (CLSM) observation. Significant colocalization could be observed in the fused group, while the mixed group overlapped only rarely (Figure 2D). Moreover, PB@LC covered with the fused materials were co-cultured with PC-3 cells, and the fluorescence of Bm and Pm overlapped well, suggesting that PB@LC was completely integrated into PC-3 cells (Figure 2E). SDS-PAGE results showed that PBm, as well as PB@LC, retained the characteristic proteins of Bm and Pm (Figure 2F). Furthermore, STRO-1 31 or cadherin-11 (CDH11) 32 was selected as a specific membrane protein marker of PC-3 or rat BMSC cells for immunogold TEM imaging (Figure 2G). The immunogold TEM images showed that immunogold-labeled STRO-1 (20 nm) and CDH11 (10 nm) could be markedly presented on PB@LC. All these results suggested that PBm was well-fused and covered on the surface of LC.

### *In vitro* cell uptake, intracellular transport and targeting effects of BmCRPC

**Figure 3:**
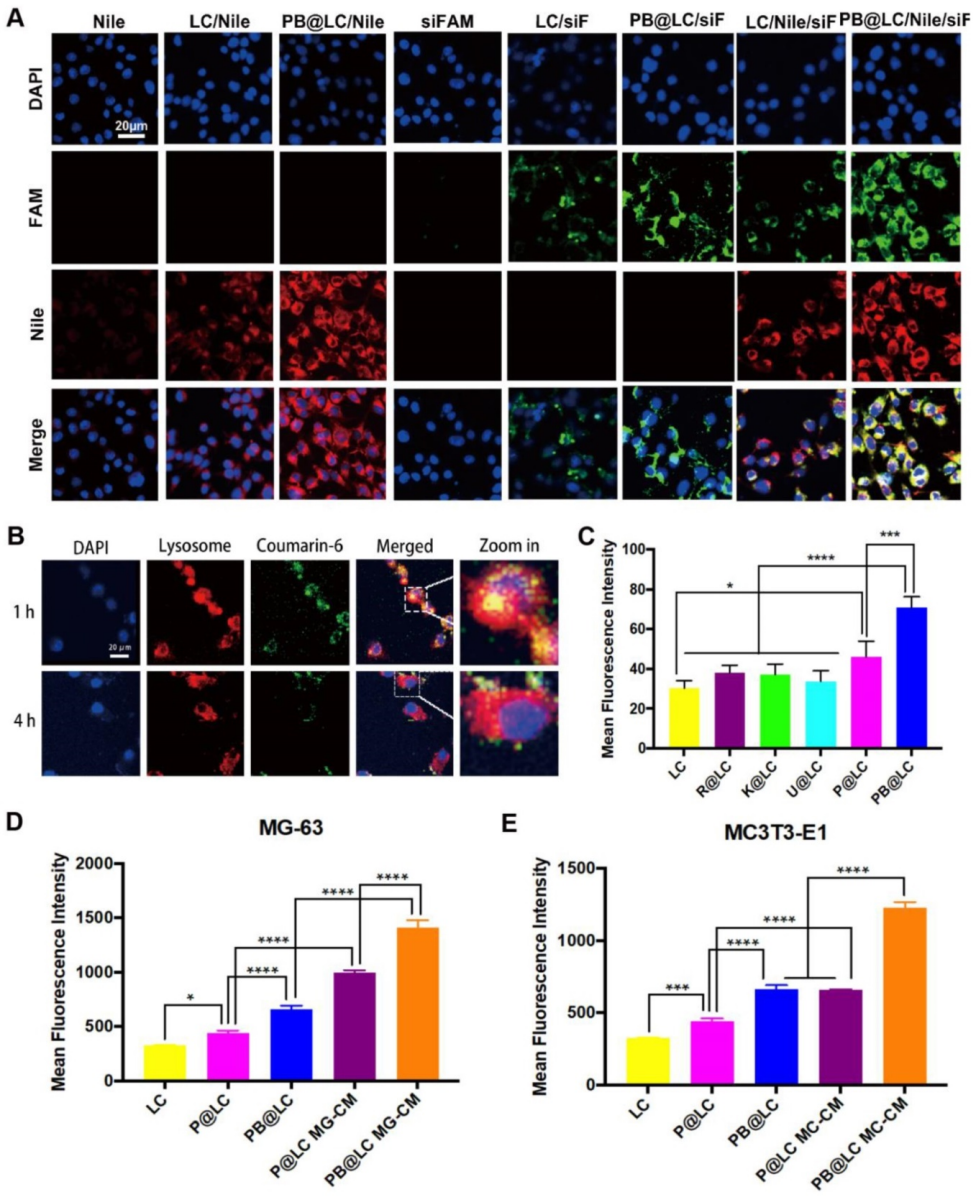
The targeting abilities of PB@LC. CLSM images of A) intracellular colocalization of each group (scale bars = 20 μm). B) The CLSM results of lysosome escape of PB@LC (scale bars = 20 μm). C) Statistic results of flow cytometry, LC was coated with different cell membranes Rm (RWPE-1), Km (KETR-3), Um (U251), Pm, and PBm (n=3, mean ± SD), *p < 0.05, **p < 0.01, ***p < 0.001, ****p < 0.0001, one-way ANOVA. D-E) The statistic results of bone targeting ability evaluation, PC-3 cells were co-incubated with P@LC and PB@LC in normal media or in conditioned media of D) MG-CM or E) MC-CM (n=3, mean ± SD), *p < 0.05, **p < 0.01, ***p < 0.001, ****p < 0.0001, one-way ANOVA.

The cell uptake of PB@LC on PCa cells was investigated by flow cytometry. Nile Red (Nile) and FAM-labeled siRNA (siFAM) were selected as model drugs. The results showed that the fluorescence of Nile or siFAM in PB@LC was 1.5-2 times as compared with that in LC, which demonstrated that PBm increased the cell uptake of LC (Figure S6). Moreover, the intracellular colocalization of the biomimetic PB@LC was investigated by CLSM to illustrate the entry of PB@LC into PCa cells. As shown in Figure 3A, much higher fluorescence intensity of PB@LC was observed than that of LC or free Nile/siFAM.

However, gene drugs are highly susceptible to phagocytic degradation by intracellular lysosomal systems. 33-35 Therefore, the endocytosis of PB@LC was tracked using Lysotracker Red to label lysosomes. Coumarin-6 was used as a model drug, and Coumarin-6 loaded PB@LC (PB@LC/C6) was prepared. A strong orange fluorescence was observed after 1 h of incubation, suggesting that PB@LC/C6 was enveloped in lysosomes. After 4 h of incubation, green fluorescence was separated from red fluorescence, indicating the liberation of PB@LC/C6 from lysosomes (Figure 3B). Our previous research also indicated that LA-NP could escape from lysosome through clathrin-mediated endocytosis and caveolae-mediated endocytosis.^15^ These results further demonstrated that PB@LC could be a highly efficient nanocarrier for gene delivery by means of lysosome escape, thereby preventing deactivation and degradation of siRNA before arriving at effect sites.

It was reported that cancer cell membranes could target allogeneic cancer by homologous targeting, while BMSC cells derived from bone marrow could home to bone. 19,36 The homologous targeting ability of PB@LC was investigated by flow cytometry with Nile Red as a model drug. LC and LC coated with various cell membranes, including human normal prostate epithelial cell RWPE-1, human renal cancer cell KETR-3, human glioma cell U251, and PCa cell membranes, were used as controls. As shown in Figure 3C, PB@LC had higher cell uptake than LC coated with other cell membranes. Moreover, the CLSM results further illustrated the homologous PCa targeting ability of our biomimetic nanoparticles (Figure S7). Additionally, to further demonstrate the bone homing ability, we constructed an *in vitro* BmCRPC model as reported by Tang *et al*.^37^ The human osteosarcoma cell line MG-63 and the mouse embryonic osteoblast cell line MC3T3-E1 were coincubated using a conditioned medium of PC-3 cells. Meanwhile, the conditioned media MG-CM (of MG-63) or MC-CM (of MC3T3-E1) were collected to mimic the microenvironment of BmCRPC. As shown in Figure 3D and E, PC-3 cells incubated with MG-CM and MC-CM had as much as a 2-fold cell uptake compared with PC-3 cells cultured in normal medium. In this study, we certified the bone-cancer dual targeting ability of PB@LC *in vitro*.

### *In vitro* anti-proliferation, invasion, metastasis and fat metabolism effects of BmCRPC

**Figure 4:**
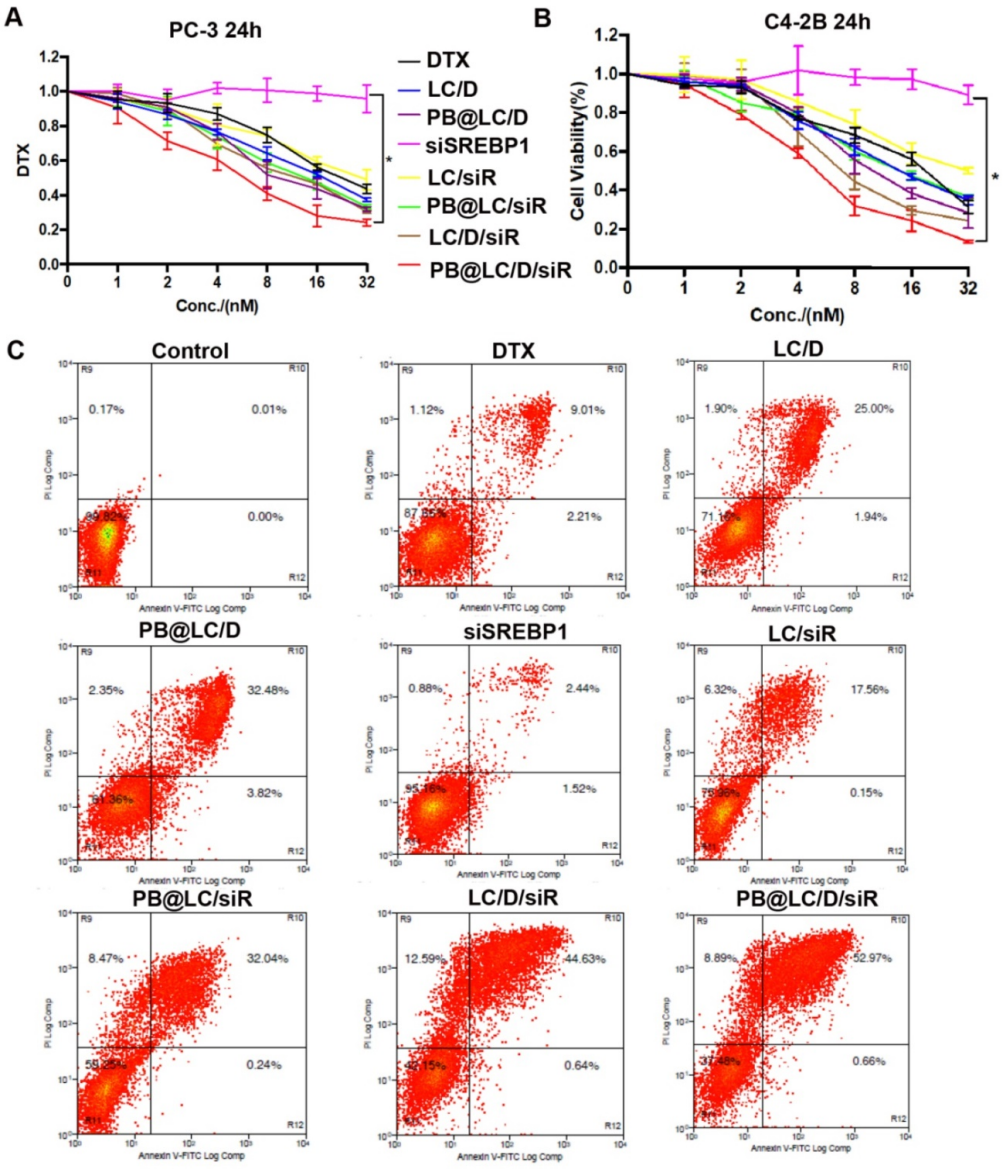
*In vitro* antiproliferation ability of PB@LC/D/siR. A-B) The cytotoxicity results of each group in A)PC-3 cells and B) C4-2B cells, the concentration gradients of DTX or siSREBP1 were 1 to 32 nM (n=3, mean ± SD). *p < 0.05, one-way ANOVA. C) The representative results of apoptosis of co-incubating PC-3 cells with each group for 24 h, DTX: 20 nM, siSREBP1: 5 nM, PBS was as control.

The* in vitro* cytotoxicity of PB@LC/D/siR on PC-3 or C4-2B cells for 24 h was evaluated. The results showed that PB@LC groups had higher *in vitro* therapeutic effects than LC groups or free drugs. Moreover, the cytotoxicity of the coloading groups was higher than that of the single drug-loading groups (Figure 4A,B). The half maximal inhibitory concentration (IC_50_) of each drug group on PC-3 cells was shown in Figure S8. As shown in Figure S8, the cytotoxicity of PB@LC/D/siR on PC-3 or C4-2B cells was 4.9 or 4.8-fold greater than that of free DTX. The IC_50_ values of naked siSREBP1 were too high and were considered to have no significance (NS). Normally, naked siRNAs have almost no pharmacological effects, since naked siRNAs are easy to be cleared by intracellular and extracellular enzymes. 38 Moreover, apoptosis results were in line with those of anti-proliferation experiments. As shown in Figure 4C, the apoptosis rates of the PB@LC groups were higher than those of the other LC groups or free drugs groups.

**Figure 5:**
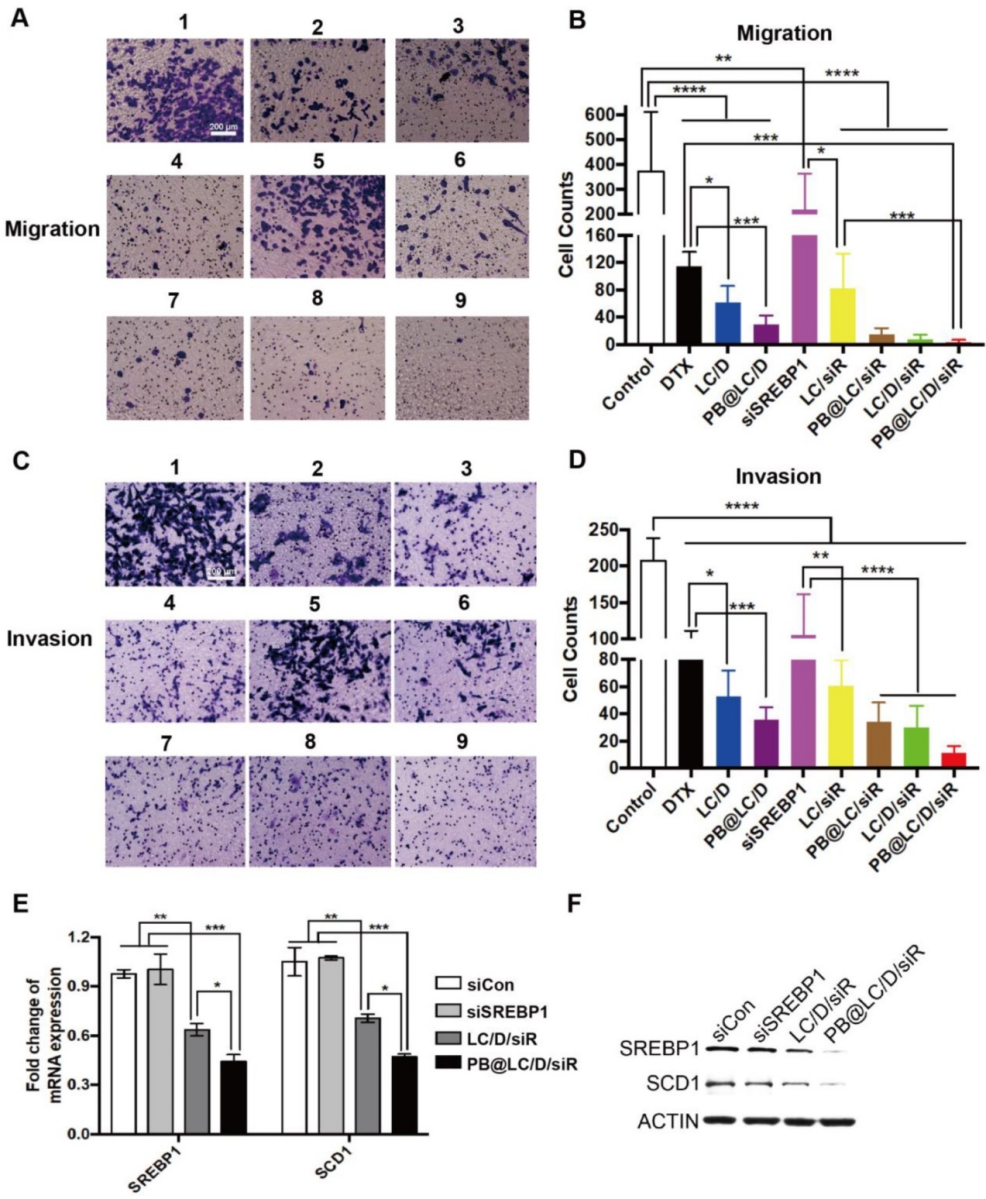
The anti-migration, anti-invasion and anti-lipogenesis assays. A) and C) PC-3 cells were incubated with each group, and 1, 2, 3, 4, 5, 6, 7, 8, 9 represented PBS (as Control), DTX, siSREBP1, LC/D, LC/siR, PB@LC/D, PB@LC/siR, LC/D/siR, and PB@LC/D/siR for 24 h or 48 h, DTX: 20 nM, siSREBP1: 5 nM, PBS was as control (scale bars = 200 μm). B) and D) The statistical analysis of cells in nine fields of view, respectively (n=9, mean ± SD). *p < 0.05, **p < 0.01, ***p < 0.001, ****p < 0.0001, one-way ANOVA. E) The RTFQ PCR results of the expression of SREBP1 and SCD1 in PC-3 cells (n = 3, mean ± SD). *p < 0.05, **p < 0.01, ***p < 0.001, one-way ANOVA. F) The Western blotting results of protein levels of SREBP1 and SCD1 in each group.

SREBP is an important gene promoting invasion and metastasis of PCa; 39 thus, the anti-invasion and anti-metastasis abilities of PB@LC/D/siR were evaluated by Transwell study. As shown in Figure 5A-D, PB@LC/D/siR exhibited the highest anti-migration and anti-invasion abilities among all the groups. There was no significant difference between the PBS and naked siSREBP1 groups due to the instability of naked siRNA. Moreover, similar effects were observed in the cell scratch test (Figure S9).

In addition, as siSREBP1 was used to prevent the progression and metastasis of PCa by interfering with the expression of SREBP1, the mRNA and protein levels of SREBP1 were evaluated. As shown in Figure 5E and F, the systemically administered PB@LC/D/siR elicited significant reductions in both the mRNA level and the protein level of SREBP1 as compared with the negative control siRNA (siCon) and naked siSREBP1. 40 Meanwhile, stearoyl-CoA desaturase 1 (SCD1) is one of the main downstream regulators of SREBP1. Both of RTFQ PCR and Western blotting results indicated the loss of SREBP1 and SCD1, which echoed the results of the reported study. 41 All of these results demonstrated the sequence-specific gene silencing efficiency of PB@LC/D/siR in BmCRPC* in vitro*.

### *In vivo* biodistribution, therapeutic and mechanism study

**Figure 6:**
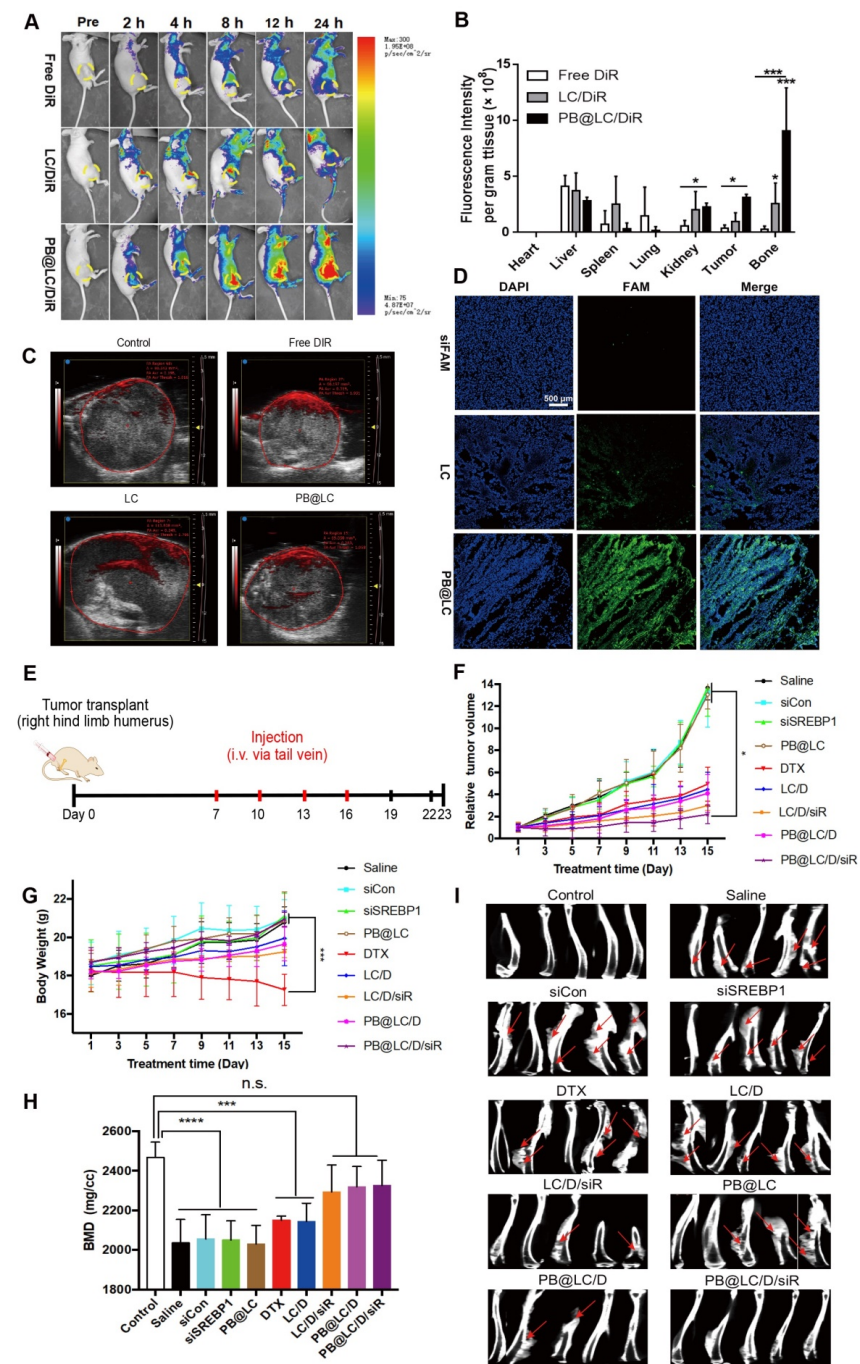
*In vivo* study of PB@LC. A) The representative small animal living images of each group of the BmCRPC-bearing mice at 0-24 h, post-injection (yellow circle: tumor area). B) The qualified distribution in major organs of each group (n=3, mean ± SD). *p < 0.05, **p < 0.01, ***p < 0.001, one-way ANOVA. C) The representative photoacoustic images of each group, saline as control. D) The CLSM images of fluorescence distribution in the tumor mass of each group, siFAM was as model drug (scale bars = 500 μm). E) The *in vivo* study protocol of PB@LC/D/siR, the concentration of siRNA or DTX was 1 mg/kg or 0.25 mg/kg, respectively. F) The tumor volume curves or G) body weight growth curves of the 9 groups (n = 5, mean ± SD). *p < 0.05, **p < 0.01, ***p < 0.001, one-way ANOVA. H) The BMD values of the BmCRPC-bearing tibias of each group, tumor-free normal tibias were as control (n = 5, mean ± SD). ***p < 0.001, ****p < 0.0001, n.s. no significance, one-way ANOVA. I) The micro-CT images of each group (n = 5, red arrows: bone damaged sites).

To investigate *in vivo* biodistribution, a BABL/c nude mouse BmCRPC model was established by injection of PC-3 cells into the long axis of the shinbone, 42 tumor formed within a week (Figure S10). PB@LC was labeled with the NIR dye DiR to trace the *in vivo* biodistribution. As shown in Figure 6A, the accumulated fluorescence at the tumor site could be monitored in the LC/DiR, PB@LC/DiR groups at 2 h early. The fluorescence intensity of LC started to decrease at 12 h, while the fluorescence intensity of PB@LC/DiR was continuously strengthened in the tumor site from 0-24 h, suggesting that PB@LC/DiR could target BmCRPC accurately with a long circulation. Figure 6B and Figure S11 showed the fluorescence biodistribution in each organ of the mice. The PB@LC/DiR group had a distinct fluorescence intensity in the tumor and bone, while the fluorescence of the free DiR group was primarily distributed in the liver and spleen, suggesting the bone-cancer dual targeting ability of PB@LC *in vivo*. Moreover, the permeability of tumor tissue is essential for solid tumor therapy. 43 The *in vivo* photoacoustic images showed that PB@LC deeply penetrated into the tumor mass with high permeability than LC, while the red fluorescence in the free DiR group was mainly distributed on the surface of the tumor mass (Figure 6C). Additionally, the promoted tumor penetration ability was confirmed by CLSM analysis of cryosections of tumors. As shown in Figure 6D, the green fluorescence intensity of siFAM in the PB@LC group was considerably stronger than LC, while no obvious fluorescence was observed in the free siFAM group. Moreover, the green fluorescence throughout the entire slice in PB@LC, indirectly proved that PB@LC had good tumor penetration ability. These results were in accordance with those of *in vitro* colocalization experiments, which showed that PB@LC could target BmCRPC accurately *in vivo*.

### *In vivo* therapeutic, anti-lipid metabolism effects and biosafety study

We further investigated the *in vivo* therapeutic effects and biosafety of PB@LC, using the BmCRPC model above. All the mice were randomly divided into 9 groups, and drugs were administered when tumors grew up to approximately 400 mm^3^ (Figure 6E). The changes in body weight and tumor volume of nude mice in each group were recorded in 15 days, and the first dose was regarded as the first day. As shown in Figure 6F, body weight of the PB@LC/D/siR group increased steadily, which was not significantly different from that of the saline, siCon, siSREBP1, and blank PB@LC groups. However, the DTX group suffered a body weight loss, which might be due to the toxicity of DTX. Meanwhile, the tumor volumes of PB@LC/D/siR-treated mice were noticeably smaller than in other groups, while the tumor volumes of the saline, siCon, siSREBP1 and PB@LC groups were continuously increased (Figure 6G). Representative tumor image of each group was presented in Figure S12.

To certificate the ability of PB@LC/D/siR in relieving bone loss in tumor-bearing tibias, microCT was applied for analysis. As shown in Figure 6H, mice treated with saline, siCon, siSREBP1 and PB@LC had suffered a great bone loss. However, the BMD values of the LC/D/siR, PB@LC/D, and PB@LC/D/siR groups were not significantly different from those of the control groups, suggesting the bone-protecting ability of PB@LC/D/siR. The microCT images of each group were shown in Figure 6I. Clearly, osteolysis could be observed in each group except the control and PB@LC/D/siR groups. These results illustrated the bone preservation ability of PB@LC/D/siR. All these results demonstrated a significant tumor inhibition ability of PB@LC/D/siR at a low concentration (DTX: 1 mg/kg) for BmCRPC with a large tumor volume (400 mm^3^).

**Figure 7:**
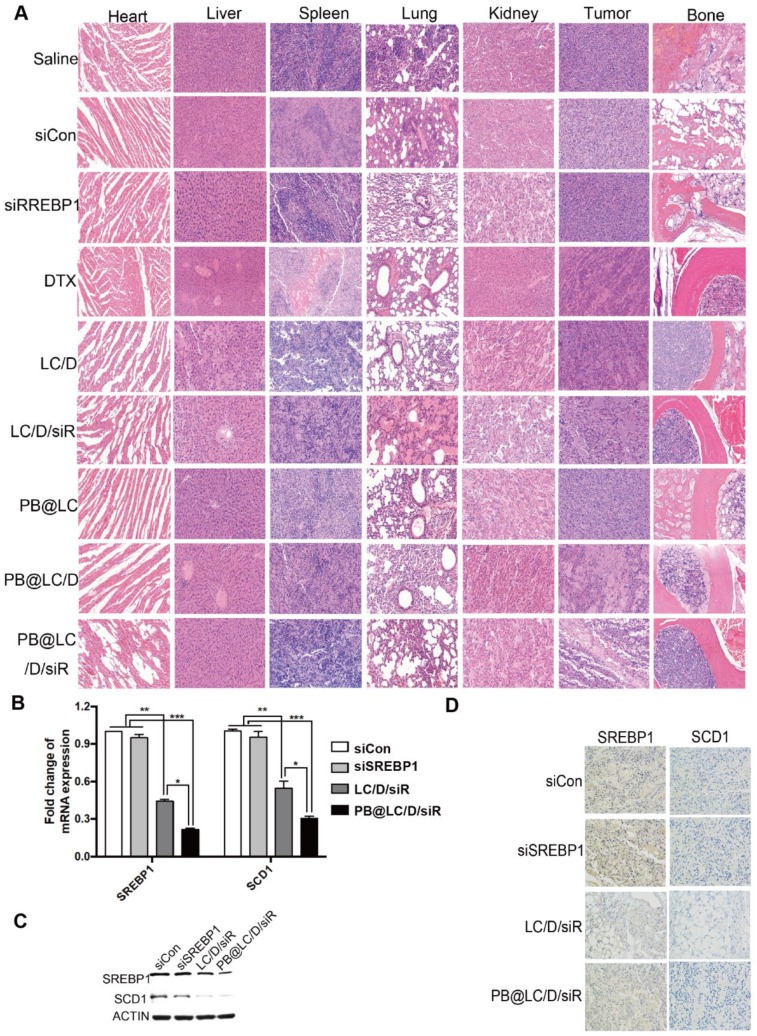
The safety and mechanism of PB@LC/D/siR. A) The HE images (200 ×) of the heart, liver, spleen, lung, kidney, tumor, bone (right hind limb tibia) of each group. B) The RTFQ PCR results of the transcription of SREBP1 and SCD1 in each group (n = 3, mean ± SD). *p < 0.05, **p < 0.01, ***p < 0.001, one-way ANOVA. C- D) The expression of SREBP1 and SCD1 in the tumor site of each group, C) Western blotting results. D) IHC images (400 ×).

In addition, the results of hematoxylin-eosin (HE) staining of major organs, tumor and bone (of the tumor site) indicated that the alleviated toxicity of DTX on normal tissues, enhanced the oncolytic effect and bone protecting effect of PB@LC/D/siR (Figure 7A). As shown in Figure 7B, an ~ 70% drop in the transcription of SREBP1 and SCD1 was detected in the PB@LC/D/siR group, which was considerably higher than in the other groups. Moreover, the protein levels of SREBP1 and SCD1 in the PB@LC/D/siR group were significantly downregulated compared with the naked siCon and siSREBP1 groups (Figure 7C). Additionally, immunohistochemistry (IHC) images (Figure 7D) showed a more intuitive reduction of SREBP1 and SCD1. These results further demonstrated the gene silencing efficiency of PB@LC/D/siR in BmCRPC* in vivo* with low toxicity.

## Discussion

Metastasis is a lethal factor in the progression of tumor, directly linked to significant lower survival rates in most cases, and once cancers metastasize to bone, skeletal related events (SREs) like considerable pain and abnormal bone remodeling occurs, resulting poor prognosis and diminished treatment outcome. 44,45 Bone is one of the most attractive site for CRPC, and MSCs are involved in the formation of the pre-metastatic niche, a forerunner of subsequent migration and evasion, continuing to deteriorate the disease. 45 However, the existing drugs showed limited alleviating effects for BmCRPC, because of side-effects and off-target. 45,46 Cell membrane-coated biomimetic nano-delivery system has evolved as a rising method for cancer-targeted delivery, with natural homologous targeting ability as well as inherited complete cell membrane protein system such as adhension factors and chemokines, which remained part of the biofunction of signaling communication. 18 We hypothesis that bone marrow derived mesenchymal stem cell membranes could target the bone metastatic site with their natural bone homing characteristics, and cell membranes derived from PCa cells could theoretically homologous target prostate cancer. As hybrid cell membranes could inherit different biofunctions from different cell resources, 47, 48 fused BMSC cell membranes with PCa cell membranes may provide a bone-cancer dual-targeting ability for drug delivery systems.

In the current study, we designed a bone-cancer dual targeting biomimetic system cloaked with a fused cell membrane of PCa cells and BMSC cells. The cell uptake in PCa cells of the established PB@LC increased compared with free model drug, and further increased when co-incubated with conditioned media-mimic of bone metastatic microenvironment. Furthermore, the *in vivo* distribution study also exhibited a good bone-cancer dual-targeting ability, with a continuously enhanced fluorescent signal in the BmCRPC site during 0-24 h, which suggested a long circulation function of PB@LC. Additionally, PB@LC also showed a good tumor-penetrating ability and was able to distribute throughout the tumor mass. These results demonstrated that the fused membranes had more advantages than singular membrane system and reserved bone homing ability of BMSC cells.

Dysregulated metabolism is another important factor leads to the progression of cancers. Cancers usually face heavy metabolic stress, with the feature of uncontrolled growth and expansion. Upregulated lipid synthesis is one means of the “cunning” cancers stocking up on carbon and energy for progression, metastasis and recolonization. 49,50 It is reported that abnormal lipid synthesis also exists in prostate cancer, promoting progression as well as metastasis, and SREBP1 is a significant regulator. 9 SREBP-dependent lipogenic program could stimulate de novo lipid biosynthesis, which contributes to the intracellular fatty acid pool of cancer majorly. 6,50 Additionally, SCD1, one of downstream gene of SREBP1, is a critical oxygen-dependent factor of the balance of cellular unsaturated fatty acids levels. The proliferation of cancer cells is considerable dependent on the SCD1-mediated supply of unsaturated fatty acids, this dependence is reduced in a hypoxic environment and restored in a low serum environment. Moreover, inhibition of SCD1 could restore the sensitivity of PCa to chemotherapeutic drugs and metabolic inhibitors. 49,50

DTX is a traditional and front-line chemotherapeutic drugs for metastatic CRPC, it is highly susceptible to drug resistance, and PI3K/AKT is one of the possible signaling pathways leading to drug resistance of DTX to prostate cancer. 10,11 Moreover, PI3K/AKT pathway has diverse downstream effects on reprogramming cellular metabolism, it could support the proliferation, survival and metastasis of cancer cells with its downstream network. 51 There are evidences that SREBP siRNAs (siSREBP1 and siSREBP2) could inhibit the progression and metastasis of PCa by repressing the PI3K/AKT signaling pathway. 8,9 Therefore, siSREBP1 could restore the sensitivity to prostate cancer of DTX and enhance the effects of reducing the progression and metastasis of prostate cancer by inhibition of PI3K/AKT when synergize with DTX.

In this study, the pharmacodynamics and mechanism experiments were linked together to verify our hypothesis that siSREBP1 could restore the sensitivity of DTX to prostate cancer, enhance the cancer suppression effects, and inhibit the progression and metastasis of prostate cancer both* in vitro* and* in vivo*. PB@LC/D/siR could inhibit the tumor growth even at a large size (400 mm^3^), and the results of micro-CT images and BMD values suggested that PB@LC/D/siR had reduced the burdens of SREs, with less bone damages. Additionally, siSREBP1 not only interfered the expression of SREBP1 but also downregulated the expression of downstream gene SCD1, which could delay the progression of BmCRPC by repressing the lipid supply.

## Conclusions

In this study, we designed a bone-cancer dual-targeting biomimetic system coated with PCa-BMSC fused cell membranes for co-delivery of DTX and siSREBP1. Supporting the utility of this natural positive targeting materials to the bone metastatic site of BmCRPC, the established PB@LC/D/siR penetrated deeply into the tumor mass and exhibited a good retention ability based on homologous targeting ability. Furthermore, the combination of SREBP1 and DTX exhibited enhanced tumor inhibition and anti-metastatic abilities by regulating abnormal lipid metabolism. Additionally, by allaying the tumor burden in the bone metastasis site, PB@LC/D/siR showed a significant bone-protecting effect without observed toxicity. This biomimetic nanosystem has expanded the means of treating BmCRPC.

## Experimental section

Full materials and methods are provided in **Supplementary Material**.

## Supplementary Material

Supplementary materials and methods, figures.

## Abbreviations

CRPC: castration-resistant prostate cancer
DTX: docetaxel
SREBP1: sterol regulatory element-binding protein 1
PCa: prostate cancer
BmCRPC: bone metastatic castration-resistant prostate cancer
LA: lipoic acid
LACL: cross-linked peptide-lipoic acid micelle
BMSC: bone marrow mesenchymal stem cell
MSCs: mesenchymal stem cells
DLS: dynamic light scattering
TEM: transmission electronic microscope
pEGFP: plasmid of enhanced green fluorescent protein
PEI: polyetherimide
SD rat: Sprague-Dawley rat
HBSS: Hank's balanced salt solution
FRET: Förster resonance energy transfer
DOPE-RhB: 1,2-dioleoyl-sn-glycero-3-phosphoethanolamine-N-(lissamine rhodamine B sulfonyl) (ammonium salt)
C6-NBD: N-[6(7-nitro-2-1,3-benzoxadiazol-4-yl)amino]hexanoyl]-D-erythro-sphingosine
PBS: phosphate buffered saline
BCA: bicinchoninic acid
SD: standard deviation
DiR: 1,1'-dioctadecyl-3,3,3',3'-tetramethylindotricarbocyanine iodide
DiO: 3,3'-dioctadecyloxacarbocyanine perchlorate
CLSM: confocal laser scanning microscope
SDS-PAGE: sodium dodecyl sulfate polyacrylamide gel electrophoresis
FAM: 5-carboxy-fluorescein
CCK-8: Cell Counting Kit-8
IC_50_: half maximal inhibitory concentration
NS: no significance
RFTQ PCR: real-time fluorescence quantitative polymerase chain reaction
SCD1: stearoyl-CoA desaturase 1
ANOVA: analysis of variance
NIR: near infrared
HE: hematoxylin-eosin
IHC: immunohistochemistry
SREs: skeletal related events
