# Supplementary material for: SREBP1 siRNA enhance the docetaxel effect based on a bone-cancer dual-targeting biomimetic nanosystem against bone metastatic castration-resistant prostate cancer
Source: Theranostics. 2020 Jan 1;10(4):1619–32. doi: 10.7150/thno.40489 (PMC6993241; doi:10.7150/thno.40489)
Supplement: Supplementary file 1 — Supplementary materials and methods, figures. [file thnov10p1619s1.pdf]

Supporting Information for

SREBP1 siRNA enhance the docetaxel effect based on a bone-cancer dual-targeting biomimetic nanosystem against bone metastatic castration-resistant prostate cancer

Jiyuan Chen[†], Zhenjie Wu[‡], Weihong Ding[§], Chengwu Xiao[‡], Yu Zhang[†], Shen Gao[‡], Yuan Gao [*,†,‡], Weimin Cai[*,†]

[†]Department of Clinical Pharmacy and Drug Administration, School of Pharmacy, Fudan University, Shanghai 201203, China

[‡]Department of Urology, Changhai Hospital, Second Military Medical University, Shanghai 200433, China

[§]Department of Urology, Huashan Hospital, Fudan University, Shanghai 200040, China

Corresponding Author

*Yuan Gao: yuan_gao@fudan.edu.cn (Y. Gao),

*Weimin Cai: weimincai@fudan.edu.cn (W. Cai)

Materials and Methods

Materials: The materials used in the synthesis of LA-NP/DTX and cross-linked peptide-lipoic acid micelle (LACL) could refer to our previous works.[1,2] DTX was purchased from BBI Life Science, China. SiSREBP1 (forward sequence: 5'-CGGAGAAGCUGCCUAUCAATT-3', reverse sequence: 5'-UUGAUAGGCAGCUUCUCCGTT-3') and negative control (labeled with 5'-FAM) (forward sequence: 5'-UUCUCCGAACGUGUCACGUTT-3', reverse sequence: 5'-ACGUGACACGUUCGGAGAATT-3') were purchased from GenePharma, China. Nile Red (Yuanyebio, China) and siFAM (GenePharma, China) were used as model drugs of DTX and siSREBP1. Phosphate buffered saline (PBS) (BasalMedia, China), 0.25% Trypsin (Gibco, USA), Hanks' Balanced Salt Solution (HBSS) (Gibco, USA), TM-Buffer (pH 7.4, + 0.01 M Tris, + 0.001 M $MgCl_2$) (Phygene, China) and lymphocyte separation solution (Dakewe Biotech, China) were purchased for cell experiments. Plasmids of enhanced green fluorescent protein (pEGFP) (Genomeditech, China), PEI (Yuanye Bio, China), 50 × TAE (Tris/Acetic Acid/EDTA) Buffer (Sangon Biotech, China), agarose (Biowest, Spain) and Gelred (Biotium, USA) were purchased for agarose gel electrophoresis. Cell Counting Kit-8 (CCK-8) (Yeasen Bio, China) and Annexin V-FITC apoptosis detection kit (Sigma-Aldrich, USA) were used for cytotoxicity and apoptosis studies. Fluorescent substances DAPI ($\lambda ex/\lambda em$ = 358/461 nm) (Yeasen Bio, China), Lysotracker Red ($\lambda ex/\lambda em$ = 577/590 nm) (Beyotime Biotech, China), Coumarin-6 ($\lambda ex/\lambda em$ = 466/504 nm) (Yuanyebio, China), DiO ($\lambda ex/\lambda em$ = 484/501 nm) (Biotium, USA), DiR ($\lambda ex/\lambda em$ = 748/780 nm) (Biotium, USA) and C6-NBD ($\lambda ex/\lambda em$ = 460/534 nm)/DOPE-RhB ($\lambda ex/\lambda em$ = 560/583 nm) (Avanti, USA) were purchased for fluorescence imaging study.

Cell lines and animals: PC-3, C4-2B, RWPE-1, HEK-293T, MG-63, MC3T3-E1, U251 and KETR-3 cells were provided by Shanghai Cell Bank, Chinese Academy of Sciences (CAS, China). PC-3, C4-2B and MC3T3-E1 cells were

cultured in Dulbecco's modified Eagle's medium (DMEM) (Gibco, USA), RWPE-1, HEK-293T, KETR-3 and U251 cells were cultured in RPMI 1640 medium (Gibco, USA), while MG-63 cells were cultured in McCoy's 5A medium (Gibco, USA). Rat BMSC cells were derived from 4-week-old SD male rats and cultured in DMEM/F12 1:1 medium (Hyclone, USA). All cell media were added with 10% fetal bovine serum (FBS) (Gibco, USA) and 100 U/mL penicillin/streptomycin (P/S) (Gibco, USA). Cells were kept at 37°C and 5% CO_2 under a constant humidity conditions.

Male SD rats (4 weeks) and male nude BALB/c mice (6 weeks) were purchased from the Second Military Medical University Animal Care Center (Shanghai, China) and were housed under specific pathogen-free (SPF) conditions. Mice were acclimatized for at least 5 days under the animal care facility. Experiments were applied under the approval of the Research Center for Laboratory Animals of the Second Military Medical University of China.

Preparation and characterization of LC and LC/D/siR. LA-NP, LA-NP/DTX, LACL micelles and LACL/siRNA micelles were prepared as previous report. [1,2] Briefly, 100 mg and 17 mg cysteine (cys) were immersed in 1 mL methanol and stirred for 8 h at room temperature (RT) to get cross-linked lipoic acid (LA). 100 mg cross-linked LA and 5 mg DTX were immersed in 1 mL chloroform and 4 mL sodium cholate (1%), sonicated at 400 W for 15 s, replicated 3 times. The obtained emulsion were added to 10 mL double-distilled (DD) water and stirred for overnight. LA-NP/DTX were purified by ultrafiltration at 3500 rpm for 10

min with 100 k MWD ultrafiltration tube. Meanwhile, a N/P ratio of 50:1 of LACL to siSREBP1 was co-incubated for 30 min at RT and purified by dialysis (MW3500). LA-NP/DTX and LACL/siSREBP1 micelles were co-incubated at RT for 30 min to obtain LC/D/siR, purified by ultrafiltration (100 k MWD). Different weight ratios of LA-NP to LACL micelles were tested to confirm the optimal ratio. LA-NP, LA micelles and LC were prepared using the same method. Moreover, the size, PDI and zeta potentials were detected by DLS, and the stability of LC was evaluated in 30 days at 4°C. The morphology of nanoparticles was observed by TEM (FEI TECNAI G^2 S-TWIN, USA). Further, the gene compression capacity of LC was estimated by agarose gel electrophoresis (DYY-6C, China), using pEGFP as a model drug.

Gene transfection assay. To evaluate the gene transfection efficiency, 2×10^5 HEK-293T cells were seeded in each well of 12-well plates for overnight. HEK-293 cells were treated with different N/P ratios of LC/pEGFP for 24 h, followed by flow cytometry detection (FACS Calibur, BD Biosciences, USA). Traditional polycationic material PEI was as a control for gene delivery vector.

Vehicle and material safety test. CCK-8 kit was used to investigate the cytotoxicity of PB@LC. PCa cells were seeded in 96-well plates at a concentration of 10^4 cells/well. PCa cells were co-incubated with a concentration gradients of vehicles of 0-400 μg/mL for 24 h. Microplate reader (Thermo, USA) was used for detection at 450 nm.

Isolation and purity detection of rat BMSC cells. [3] For extraction of BMSC cells, 4-week old SD male rats were sacrificed and immersed in 75% alcohol for 10 s. The tibias and femurs were dissected, and washed with HBSS. Bones were cut into small pieces and bone marrow was blown out with 1-mL syringes until the bones became whitened. Then, the suspensions were gathered and centrifuged for 5 min at 1000 rpm. The collected cells were resuspended with 5 mL HBSS and transferred into a 15 mL centrifuge tube with 5 mL lymphocyte separation solution to centrifuge for 25 min at 2000 rpm. Bone marrow stromal cells were collected and seeded in T25 flasks at a concentration of 10^6 cells/mL. After 48 h of incubation, the BMSCs were obtained by removing the unattached cells. In this experiment, the third generation of BMSC cells was used for purity detection. [4] Briefly, PE-labeled rat BMSC negative markers anti-CD34 (Invitrogen, 12-0349-41, USA), anti-CD45 (Invitrogen, 12-0461-80, USA), positive markers anti-CD73 (Invitrogen, 4344363, USA), anti-CD90 (Invitrogen, Lot#2036634, USA) and anti-CD105 (Invitrogen, Lot#1983608, USA) were incubated with BMSC cells on a horizontal shaker (QiTe, STS-3, China) respectively. After a 30-min incubation at RT, the BMSC cells were harvested for flow cytometry detection.

Cell membrane extrusion and evaluation. [5] PCa or BMSC cells were collected and resuspended in 4 °C precooled Tris buffer. Then, the cells were continuously extruded back and forth by mini extruder (25 mm) (Changsha Nayi Instrument Technology Co., Ltd., China) for 20 times followed by ultrasound (500 W, 10 kHz) (Kunshan Ultrasonic Instruments Co. Ltd., KQ-500E ultrasonic cleaner,

China) for 5 min. Cellular content was removed by centrifugation at 2000 g for 10 min at 4°C (Eppendorf, Centrifuge 5804R, Germany). The supernatants were collected and centrifuged at 10,000 g for 30 min at 4°C. Cell membrane precipitation was washed and resuspended with TM-Buffer containing 0.25 M sucrose (SinoPharm, China) for centrifugation at 10,000 g for 30 min at 4°C. The zeta potential was evaluated by DLS system, and the total membrane proteins were detected by BCA method. Cell membranes were stored at -80°C for further use.

Preparation of PCa-BMSC fused membrane (PBm). Pm and Bm were obtained through methods of Zhang *et al.* [6] To obtain the optimal ratio of the Pm to Bm, we prepared a pair of Förster resonance energy transfer (FRET) dyes C6-NBD /DOPE-RhB. Different membrane protein weight ratios (Pm : Bm) of 0:1, 1:1, 2:1, 3:1, 4:1, and 5:1 were tested by fluorescence spectrophotometry (Hitachi, F-7000, Japan). Furthermore, the colocalization of Pm and Bm was evaluated by CLSM. One hundred microliters of Pm or Bm was dyed with 5 μL of 10 μg/mL green fluorescent dye DiO or red fluorescent dye DiR. The two stained cell membranes were mixed to obtain the mixed membrane or fused (ultrasound for 5 min) to obtain the fused membrane. These cell membrane materials were analyzed by CLSM.

Preparation and characterization of PB@LC. One milligram (membrane protein) of PBm was added to 1mL LC (1 mg/mL) to obtain PB@LC by ultrasound for 5 min. Then, PB@LC were resuspended in PBS at a membrane

protein concentration of 0.2 mg/mL. To assess the best weight ratio of LC to PBm, different ratios (1:0, 1:10, 1:5, 1:2, 1:1 2:1, 4:1, 5:1, 6:1, 10:1, 0:1) were examined by DLS system for optimal size and zeta potential. PB@LC was purified as reported. [1] The coverage rate of PB@LC were evaluated through BCA method. Moreover, the morphology of PB@LC was detected through TEM imaging. To assay the fusion by CLSM, LC was coated with DiO/DiR stained PBm, while the nuclei were stained with DAPI. For more accurate measurement, cell membrane-specific markers of BMSCs (STRO-1) and PC-3 (CDH11) were selected for immunogold TEM study. [6] Gold conjugated secondaries against mouse IgG (10 nm, Abcam, ab 27241, UK) and rabbit IgG (20 nm, Abcam, ab 27237, UK) to stain anti-STRO-1 (eBioscience, 14-6688-80, USA) or anti-CDH11 (R&D, MAB1790-SP, USA). The total membrane proteins were characterized by SDS-PAGE gel electrophoresis.

***In vitro* cell uptake study.** To evaluate the cell uptake. Nile Red and siFAM were used as model drugs at a concentration of 20 ng. PC-3 or C4-2B cells were seeded in 12-well plates at a concentration of 3×10^5 cells/well for overnight. Cells were co-incubated with each group for 1-4 h, simultaneously the medium of each group was changed with serum-free medium. The fluorescence intensity of each group was detected by flow cytometry.

Lysosome escape assay.[7] Cells were seeded in 24-well plates at a concentration of 10^5 cells/ well for overnight. PB@LC was loaded with Coumarin-6 (20 ng/mL) and lysosome was stained with Lysotracker Red (50 ng/mL) for 30 min.

CLSM images of 1 and 4 h were obtained to reveal the transport mechanism of PB@LC.

Homologous targeting assay. [8] LC were coated with cell membranes of RWPE-1 (Rm), KETR-3 (Km), U251 (Um), Pm and PBm. PC-3 or C4-2B cells were seeded in 12-well plates at a concentration of 3×10^5 cells/ well for overnight. After incubation with PCa cells for 2 h, the fluorescence intensity of each group was tested by flow cytometry.

Establishment of *in vitro* bone metastasis model and validation of bone targeting. The *in vitro* bone metastasis model was established as the method of Tang *et al.* [9] First, the human osteosarcoma cell line MG-63 and the mouse embryonic osteoblast cell line MC3T3-E1 were coincubated using a conditioned medium of PC-3 cells. Second, the coincubated conditioned media were further collected followed by 3 weeks of culture of MG-63 or MC3T3-E1 cells in those coincubated conditioned media. Third, the conditioned media MG-CM (of MG-63) or MC-CM (of MC3T3-E1) were collected to obtain a mimic microenvironment of BmCRPC. Then, PC-3 cells were cultured in the media with a mimic microenvironment for further investigation, and PC-3 cells cultured in normal medium without a mimic microenvironment were used as a control.

Cell anti-proliferation and apoptosis assays. PC-3 or C4-2B cells were seeded in 96-well plates at a concentration of 10^4 cells/ well overnight. A CCK-8 kit was used to evaluate the cytotoxicity of each group, as measured at 450 nm by a microplate reader. Concentration gradients of DTX or siSREBP1 were 1 to 32

nM. PC-3 cells were seeded in 12-well plates at a concentration of 3×10^5 cells/ well for overnight. An Annexin V-FITC apoptosis detection kit was used to test the apoptosis of PC-3 cells by flow cytometry with a concentration of 10/2.5 nM of DTX/siSREBP1.

Cell scratch test. To form cell scratch, culture-inserts (Ibidi, Germany) were stuck on 12-well plates, 70 μL cell suspension contained 10^5 PC-3 cells were added into each well of culture-inserts. The culture-inserts were removed until the cells were attached on the plates. Then, PC-3 cells were co-cultured with PB@LC/D/siR and other groups. Fluorescence microscope (Olympus IX78, Japan) images of each group of bright field were shot at 0, 6, 12 and 24 h .

Cell anti-migration and anti-invasion assays. A 100-μL cell suspension containing 10^5 PC-3 cells in DMEM medium without FBS was inoculated in the upper chamber of 8-μm Transwell and coincubated with each group with or without Matrigel-coated Transwell inserts for invasion or metastasis study. Meanwhile, 800 μL DMEM added with 20% FBS as chemokines was added to the lower chamber of Transwell. All the groups were incubated at a DTX/siSREBP1 concentration of 20/5 nM. After incubation for 24 h (for migration study) or 48 h (for invasion study), the surface cells in the upper chamber were wiped with a sterile cotton swab. Migrated cells were fixed in methanol (TEDIA, Lot17055009, USA) for 30 min, then the cells were stained with 0.1% crystal violet (Yuanye Bio, China) for 20 min, and cells were washed

with PBS 3 times. Nine fields of view of the images in bright field were obtained by fluorescence microscopy for statistical analysis.

Establishment of BALB/c nude mouse BmCRPC model. The BmCRPC model was established as the reported method. [10] Four-week-old male BALB/c nude mice had adapted the new environment for 1-2 weeks. The femur and tibia of the right hind limb were bent at an angle of 90°, and 5×10^5 PC-3 cells were injected by puncturing into the bone marrow cavity along the long axis of the tibia for each mouse. Tumors formed within one week.

***In vivo* fluorescence biodistribution study.** The BmCRPC animal model was established as mentioned above. DiR, LC/DiR and PB@LC/DiR were injected *via* the tail vein at a concentration of 1 mg/kg. The mice were scanned at 0, 2, 4, 8, 12 and 24 h using a Bio-Real Quick View 3000 imaging system (Bio-Real Sciences, Australia) at 655/716 nm. Then, the mice were sacrificed at 24 h, and the heart, liver, spleen, lung, kidney, tumor and bone (tibia and femur of right hind limb) were harvested with the fluorescence in each sample measured using an *in vivo* imaging system. All the data were analyzed using Quick View 3000 software.

***In vivo* photoacoustic and CLSM imaging.** [11] DiR was used as a model drug for tumor penetration study by photoacoustic imaging. Saline, free DiR, LC/DiR and PB@LC/DiR were injected in the tail vein after tumor model was established (DIR: 1 mg/kg). Penetration depth was measured by a multimode ultrasound/photoacoustic imaging system (Fujifilm VisualSonics, VEVO LAZR-

X, USA) after 4 h of injection. Additionally, siFAM, LC/siF and PB@LC/siF were injected in the tail vein at a concentration of 20 μg siRNA for each mouse. Mice were sacrificed 4 h later to prepare the cryosections of tumors. The slices were stained with DAPI for later CLSM imaging tests.

***In vivo* antitumor effects of BmCRPC.** The BmCRPC animal model was established as mentioned above. Mice were randomly divided into 9 groups: saline, siCon, siSREBP1, DTX, LC/D, LC/D/siR, PB@LC, PB@LC/D and PB@LC/D/siR when tumors grew up to approximately 400 mm^3 (n = 5). The first dose was recorded as the first day and then administered every three days for 4 times. The concentration of DTX or siSREBP1 was 1 or 0.25 mg/kg. Body weight and tumor size were measured every two days. All the mice were sacrificed on the 16th day. Tumor size was calculated using the following equation (l was the greatest diameter of the tumor, and s was the smallest).

$$\mathrm{V}\,(mm^3) = \frac{l}{s^2} \times 2$$

***In vivo* bone protection study.** The morphological change and bone density of tumor-bearing tibias were monitored using microCT (Siemens Inveon microCT, SD_000_N8-875, Germany) under 80 kVp condition with normal left hind limbs as controls (n = 5). Meanwhile, measured heat unit (HU) values were regulated by the BMD standard line of tissue-equivalent phantom (Siemens, Germany) to determine bone density (mg/cc).

Mechanism study. [12] To confirm the biofunction of siSREBP1, mRNA and protein levels of SREBP1 and SCD1 were measured through real-time fluorescence quantitative PCR (RTFQ PCR) and Western blotting in PC-3 cells (10^7) and tumor tissues (100 mg). PC-3 cells were treated with siCon, siSREBP1, LC/D/siR and PB@LC/D/siR for 48 h, the concentration of siRNA was 10 μg/mL. Tumor tissues were collected on the 16th day of *in vivo* efficacy study. RNA of cells or tissues was extracted by RNA kit (Servicebio, G3013, China) and cDNA was synthesized with RevertAid First Strand cDNA Synthesis Kit (Thermo, #K1622, USA). And FastStart Universal SYBR Green Master (Rox) (Roche, 04913914001, Switzerland) was applied for RTFQ PCR analysis by Real-Time PCR System (ABI, 7300, USA). Primers of SREBP1(Biocience, NM_001005291, China): forward sequence: 5′- TGACCCGGCTATTCCGTGA -3′, reverse sequence: 5′-CTGGGCTGAGCAATACAGTTC-3′ , SCD (Shycbio, NM_009127.4, China): forward sequence: 5′-TTCCTACCTGCAAGTTCTACACC-3′, reverse sequence: 5′-CCGAGCTTTGTAAGAGCGGT-3′ and GAPDH (Shycbio, NM_008084.2, China) : forward sequence: 5’-CCTCGTCCCGTAGACAAAATG-3’, reverse sequence: 5’- TGAGGTCAATGAAGGGGTCGT-3’, were applied for RTFQ PCR analysis. Tissue and cell lysates were prepared with RIPA buffer (Beyotime, G2002, China) and 50 × cocktail (Beyotime, G2006, China). Anti-SREBP1 (Fitzgerald, 70R-12227, USA) and Anti-SCD1 (Abcam, ab236868, UK), Anti-beta Actin Mouse mAb (Servicebio, GB12001, China), Anti-GAPDH Mouse mAb (Servicebio, GB12002, China), Anti-Histone H3.3 Rabbit pAb (Servicebio,

GB11026, China), HRP-labeled Goat Anti-Rabbit IgG (H+L) (Servicebio, GB23303, China), HRP-labeled Donkey Anti-Goat IgG (H+L) (Servicebio, GB23404, China), HRP-labeled Goat Anti-Mouse IgG (H+L) (Servicebio, GB23401, China), HRP-labeled Goat Anti-Rat IgG (H+L) (Servicebio, GB23402, China) were used for Western blotting study. Furthermore, immunohistochemistry (IHC) slices of tumor tissues of siCon, siSREBP1, LC/D/siR and PB@LC/D/siR were prepared to evaluate the expression levels of SREBP1 and SCD1 more intuitively.

***In vivo* biosafety evaluation.** The heart, liver, spleen, lung, kidney, tumor and right hind limb tibia of each mouse were harvested for H&E staining to investigate the biosafety of PB@LC/D/siR.

Statistics analysis. All numerical data were analyzed with Prism 7.0 software, mean ± SD, and at least tropical samples. The comparison of each group was performed by one-way ANOVA, while a P value < 0.05 was considered to be statistically significant.

A

	Size/nm	PDI	Zeta/mV
LC	81.0±0.5	0.179	26.9±0.1
LC/D/siR	84.4±0.3	0.120	20.3±0.1
PB@LC	92.7±1.0	0.102	-22.0±1.6
PB@LC/D/siR	100.2±0.2	0.220	-20.2±0.5

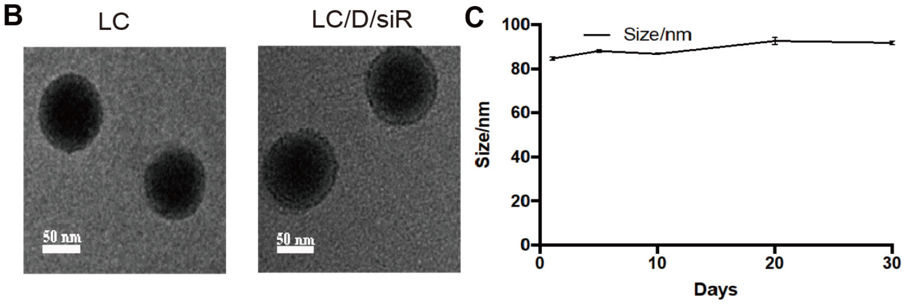

Figure S1. The characterization of nanoparticles. A) The particles size distribution and zeta potentials of LC, LC/D/siR, PB@LC and PB@LC/D/siR. B) The typical TEM images of LC and LC/D/siR (scale bars = 50 nm). C) The stability of LC in 30 days (n=3, mean ± SD).

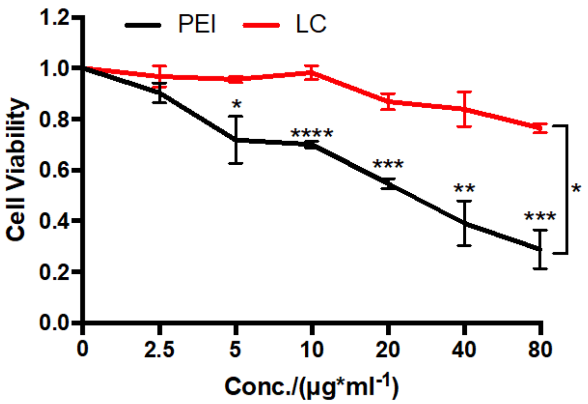

Figure S2. The CCK-8 cytotoxicity test results of PEI, LC for coincubating 24 h with HEK-293T cells, the concentration gradients of PEI or LC were 2.5, 5, 10, 20, 40 and 80 μg/mL (n=3, mean ± SD). *p < 0.05, **p < 0.01, ***p < 0.001, one-way ANOVA.

A

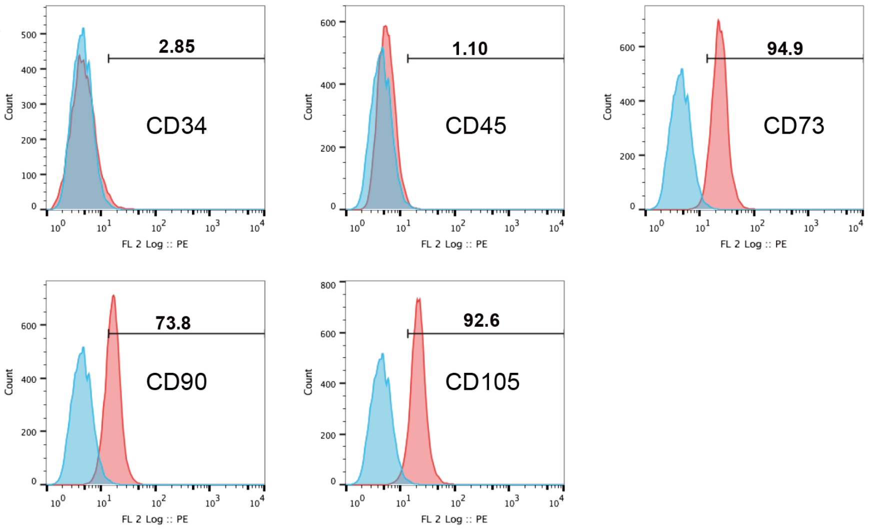

B

Cell Line	Zeta/mV	Membrane Protein (10^7 cells/mg)
Rat BMSC	-22.8 ± 0.6	0.61
PC-3	-22.2 ± 1.0	0.65
C4-2B	-22.8 ± 0.3	0.56

Figure S3. The characterization of BMSC and PCa cells. A) The flow cytometry results of the purity of SD rat BMSC cells at P3, marked with PE-labeled BMSC negative markers anti-CD34 and anti-CD45, and BMSC positive markers anti-CD73, anti-CD90 and anti-CD105. B) The zeta potentials and the containing membrane proteins of BMSC, PC-3, and C4-2B cell membranes (n=3, mean ± SD).

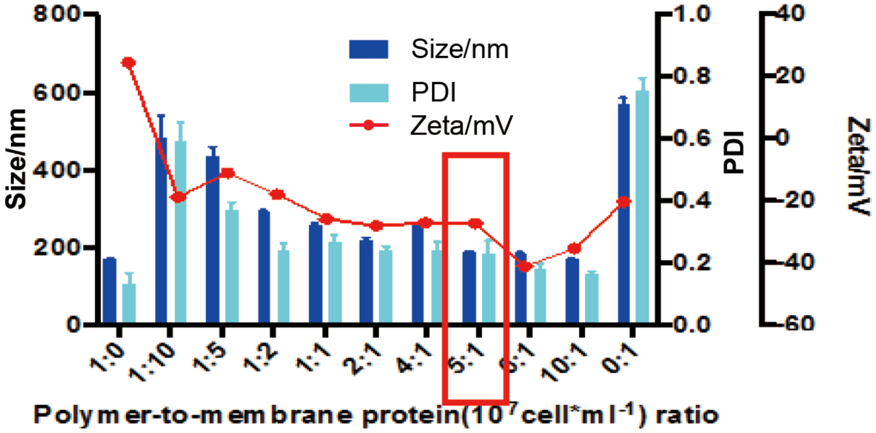

Figure S4. Verification of the optimal weight ratio of polymer to membrane protein. The size, PDI and zeta potential results of PB@LC at LC to PBm weight ratios of at 1:0, 1:10, 1:5, 1:2, 1:1, 2:1, 4:1, 5:1, 6:1, 10:1 and 0:1 (n=3, mean ± SD).

A

Sample	Protein/(mg/10^8cell)	Coverage Rate/%
PC-3 Membrane	0.65	91
P@LC	0.59	
C4-2B Membrane	0.56	88
P@LC	0.49	

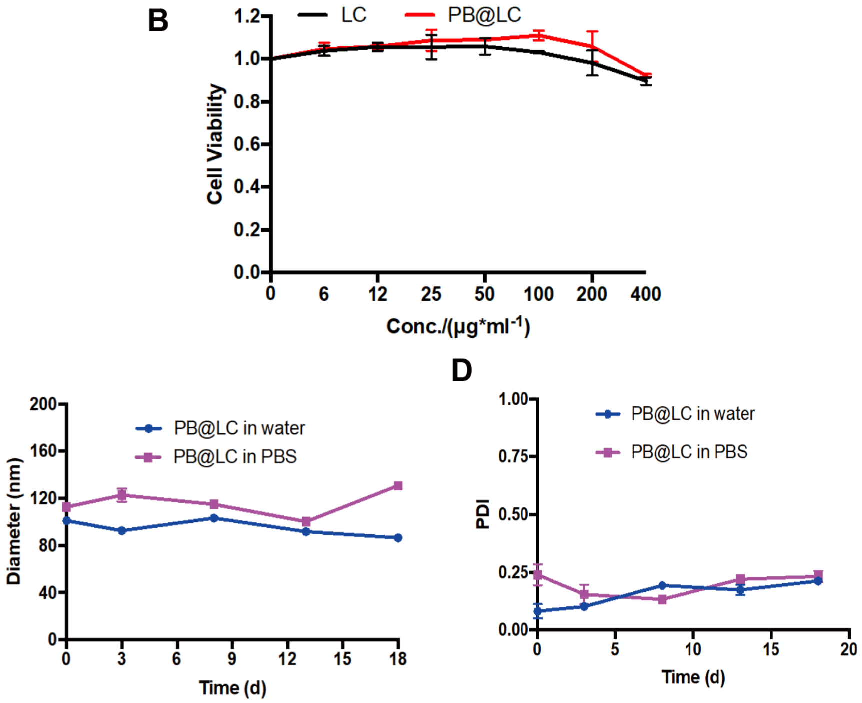

Figure S5. A) The membrane coverage rate of P@LC. B) The cytotoxicity of carriers LC and PB@LC (n=3, mean ± SD). C-D) The stability of PB@LC in water or 1 × PBS in 18 days (n=3, mean ± SD).

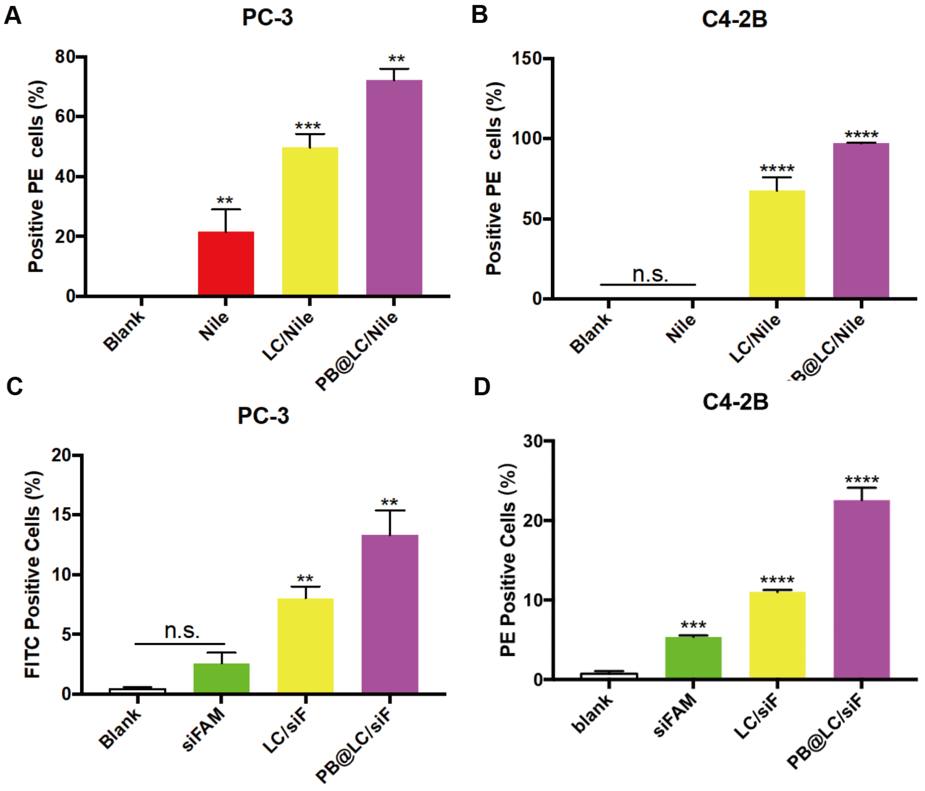

Figure S6. The cell uptake ability of PB@LC, Nile Red or siFAM was used as a model drug. A-B) Chemotherapeutic drug and C-D) siRNA cell uptake abilities of PC-3 or C4-2B cells (n=3, mean ± SD). *$p < 0.05$, **$p < 0.01$, ***$p < 0.001$, ****$p < 0.0001$, n.s., no significant, one-way ANOVA.

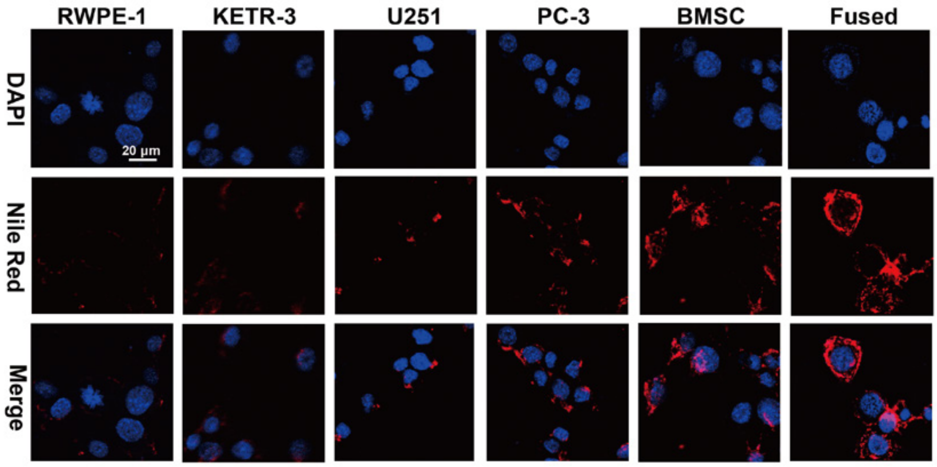

Figure S7. The CLSM images of PC-3 cells, which were co-incubated with RWPE-1, KETR-3, U251, PC-3, BMSC cell membranes and PBm coated LC/Nile for 1 h (scale bars = 20 μm).

A

Group	IC_{50}(nM)
DTX	20.1
LC/D	10.4
PB@LC/D	7.5
siSREBP1	NS
LC/siR	12.3
PB@LC/siR	8.3
LC/D/siR	6.8
PB@LC/D/siR	4.1

B

Group	IC_{50}(nM)
DTX	22.8
LC/D	9.6
PB@LC/D	7.5
siSREBP1	NS
LC/siR	12.1
PB@LC/siR	8.2
LC/D/siR	6.5
PB@LC/D/siR	4.8

Figure S8. The IC_{50} values of each group, in A) PC-3 cells and B) C4-2B cells (NS: no significance).

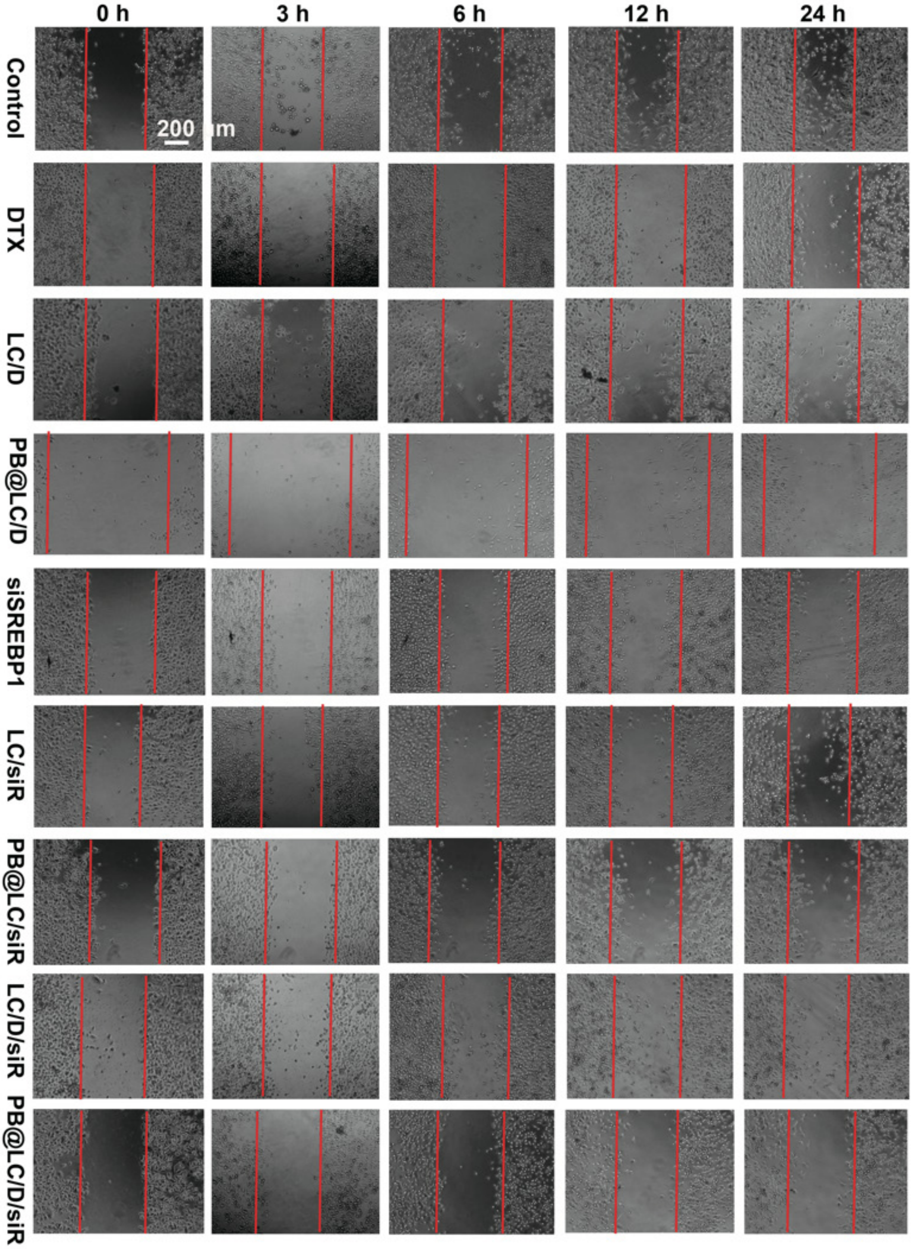

Figure S9. The representative images of cell scratch test of each group at 0, 3, 6, 12 and 24 h (scale bars = 200 μm).

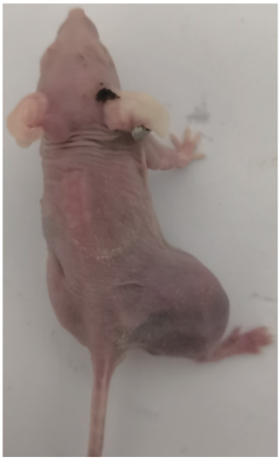

Figure S10. The formation of BmCRPC in right hind limb tibia BALB/c nude mouse.

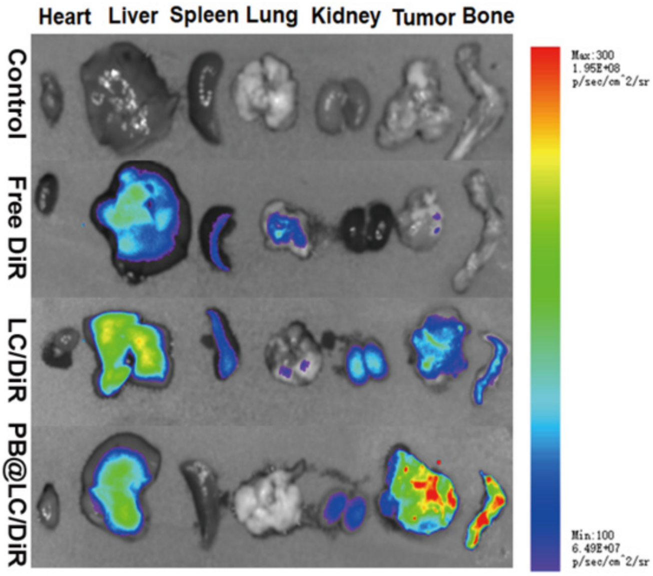

Figure S11. The typical *ex vivo* images of heart, liver, spleen, lung, kidney, tumor, bone (right hind limb tibia) of each group at 24 h after injection.

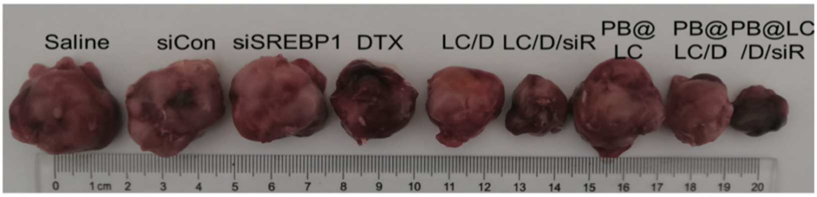

Figure S12. The representative image of excised tumor tissues of each group.

References

1. Gu F, Hu C, Tai Z, Yao C, Tian J, Zhang L, et al. Tumour microenvironment-responsive lipoic acid nanoparticles for targeted delivery of docetaxel to lung cancer. Sci Rep. 2016; 6: 36281.

2. Gong C, Hu C, Xia Q, Yao C, Zhang L, Qiang L, et al. Co-delivery of autophagy inhibitor ATG7 siRNA and docetaxel for breast cancer treatment. J Control Release. 2017; 266: 272-86.

3. Dai ZQ, Wang R, Ling SK, Wan YM, Li YH. Simulated microgravity inhibits the proliferation and osteogenesis of rat bone marrow mesenchymal stem cells. Cell Proliferat. 2007; 40; 671-84.

4. Mahboudi H, Kazemi B, Soleimani M, Hanaee-Ahvaz H, Ghanbarian H, Bandehpour M, et al. Enhanced chondrogenesis of human bone marrow mesenchymal stem cell (BMSC) on nanofiber-based polyethersulfone (PES) scaffold. Gene. 2018; 643: 98-106.

5. Cao HQ, Dan ZL, He XY, Zhang ZW, Yu HJ, Yin Q, et al. Liposomes coated with isolated macrophage membrane can target lung metastasis of breast cancer. ACS Nano. 2016; pp 7738-48.

6. Dehaini D, Wei X, Fang RH, Masson S, Angsantikul P, Luk BT, et al. Erythrocyte–platelet hybrid membrane coating for enhanced nanoparticle functionalization. Adv Mater. 2017; 29: 1-8.

7. Hu QY, Qian CG, Sun WJ, Wang JQ, Chen ZW, Bomba HN, et al. Engineered nanoplatelets for enhanced treatment of multiple myeloma and thrombus. Adv Mater. 2016; 28: 9573-80.

8. Sun HP, Su JH, Meng QS, Yin Q, Chen LL, Gu WW, et al. Cancer cell membrane-coated gold nanocages with hyperthermia-triggered drug release and homotypic target inhibit growth and metastasis of breast cancer. Adv Func Mater. 2017; 1604300: 1-9.

9. Chang AC, Chen PC, Lin YF, Su CM, Liu JF, Lin TH, et al. Osteoblast-secreted WISP-1 promotes adherence of prostate cancer cells to bone via the VCAM-1/integrin A4B1 system. Cancer Lett. 2018; 426: 47-56.

10. Hoang B, Ernsting MJ, Tang WS, Bteich J, Undzys E, Klyoto T, et al. Cabazitaxel-conjugated nanoparticles for docetaxel-resistant and bone metastatic prostate cancer. Cancer Lett. 2017; 410: 169-79.

11. Tan T, Wang H, Cao HQ, Zeng LJ, Wang YQ, Wang ZW, et al. Deep tumor-penetrated nanocages improve accessibility to cancer stem cells for photothermal-chemotherapy of breast cancer metastasis. Adv Sci. 2018; 5: 1801012.

12. Li XY, Chen Y-T, Josson S, Mukhopadhyay NK, Kim J, Freeman MR, et al. MicroRNA-185 and 342 inhibit tumorigenicity and induce apoptosis through blockade of the SREBP metabolic pathway in prostate cancer cells. PLoS One. 2013; 8: e70987.
